# 

**Published:** 1914-04-15

**Authors:** 


### ANNOUNCEMENTS.

### New Jersey State Board of Dental Examiners.

The New Jersey State Board of Dental Examiners will hold their regular meeting and examination in the Assembly Chamber of the State House, Trenton, N. J., on June 29, 30, and July 1, 1914. License fee, $25.00. No interchange of license. Applications must be filed *complete* at least ten days before date set for examination.

All applicants for a license to practise dentistry in New Jersey "shall present to said Board a certificate from the Superintendent of Public Instruction showing that before entering a dental college, he or she had obtained an academic education consisting of a four years' course of study in an approved public or private high school, or the equivalent thereof." Therefore, the Secretary will issue application blanks to applicants *only* upon the presentation of the required dental certificate from the Supt. of Public Instruction, Trenton, N. J.

A bridge, consisting of three or more teeth exclusive of abutments, and one Richmond Crown, metallic parts in gold, will be required, mounted and articulated, as a practical test in Prosthetic Dentistry, in place of a full set of teeth soldered upon a gold or coin silver plate hitherto required.

For further particulars, apply to Alphonso Irwin, D.D. S., Sec'y., 425 Cooper Street, Camden, N. J.

### National Dental Protective Association.

The Annual Meeting of this Organization will be held at the Dental Department of George Washington University in Washington, D. C., May 19th, 1914, at 7:30 P. M., for the election of trustees and officers for the ensuing year and the transaction of other business.

E. P. Dameron, President,
M. F. Finly, Secretary.

